# Endovascular Treatment of Intracranial Aneurysm: The Importance of the Rheological Model in Blood Flow Simulations

**DOI:** 10.3390/bioengineering11060522

**Published:** 2024-05-21

**Authors:** Maria Antonietta Boniforti, Giorgia Vittucci, Roberto Magini

**Affiliations:** Department of Civil, Building, and Environmental Engineering, Sapienza University, 00184 Rome, Italy; vittucci.1811256@studenti.uniroma1.it (G.V.); roberto.magini@uniroma1.it (R.M.)

**Keywords:** hemodynamics, intracranial aneurysm, flow diverter stent (FDS), non-Newtonian blood flow, oscillatory shear index (OSI), endothelial cell activation potential (ECAP), relative residence time (RRT), CFD

## Abstract

Hemodynamics in intracranial aneurysm strongly depends on the non-Newtonian blood behavior due to the large number of suspended cells and the ability of red blood cells to deform and aggregate. However, most numerical investigations on intracranial hemodynamics adopt the Newtonian hypothesis to model blood flow and predict aneurysm occlusion. The aim of this study was to analyze the effect of the blood rheological model on the hemodynamics of intracranial aneurysms in the presence or absence of endovascular treatment. A numerical investigation was performed under pulsatile flow conditions in a patient-specific aneurysm with and without the insertion of an appropriately reconstructed flow diverter stent (FDS). The numerical simulations were performed using Newtonian and non-Newtonian assumptions for blood rheology. In all cases, FDS placement reduced the intra-aneurysmal velocity and increased the relative residence time (RRT) on the aneurysmal wall, indicating progressive thrombus formation and aneurysm occlusion. However, the Newtonian model largely overestimated RRT values and consequent aneurysm healing with respect to the non-Newtonian models. Due to the non-Newtonian blood properties and the large discrepancy between Newtonian and non-Newtonian simulations, the Newtonian hypothesis should not be used in the study of the hemodynamics of intracranial aneurysm, especially in the presence of endovascular treatment.

## 1. Introduction

An intracranial aneurysm (IA) is a pathological dilatation of the vascular wall frequently observed in curves or arterial bifurcations in the Circle of Willis. It has an incidence of approximately 3% in the adult population [1]. The most serious consequence of the presence of an intracranial aneurysm is its rupture and the consequent subarachnoid hemorrhage that is associated with a high mortality and morbidity rate [2]. Interestingly, IAs are typically asymptomatic until rupture, and many intracranial aneurysms never rupture. Therefore, the risks and benefits of surgical treatment should be carefully considered.

Currently, the techniques to manage cerebral aneurysms are based on surgical or endovascular treatments. Among the less invasive treatments, the endovascular technique with flow diverter stent (FDS) deployment is increasingly used, especially for the treatment of complex, wide-neck, and giant aneurysms. The flow diverter stent is composed of a large number of braided wires, and is usually placed in the parent artery at the level of the aneurysmal neck. Its primary function is to redirect the blood flow towards the parent artery flow direction. The implantation of the stent reduces blood inflow in the aneurysmal dilation, involving a progressive exclusion of the aneurysm from the cerebral blood flow and favoring progressive thrombosis of the aneurysm. Fundamental to the effectiveness of the FDS is its porosity that is defined as the ratio between the metal-free surface and the total surface area of the stent, and has optimal values around 65–70% [3,4]. An increase in the density of the mesh of the stent determines a reduction in its porosity, thus decreasing blood flow into the aneurysm and favoring intra-aneurysmal thrombosis [5]. However, low values of porosity can increase the stiffness of the device, making its deployment difficult. Furthermore, length and pore sizes of FDS can change depending on the size and curvature of the artery where it is deployed. Then, the porosity of the FDS could be locally changed from the nominal one due to device deformation, affecting aneurysm occlusion rate [6].

Evaluating the risk of aneurysm rupture and the effectiveness of the FDS implantation is essential to help physicians assess whether the rupture risk justifies the risks associated with the treatment. For this purpose, computational fluid dynamics (CFD) investigations have proven to be a very useful tool, as the evaluation of intra-aneurysmal hemodynamics allows us to predict the evolution of the aneurysm and select the best endovascular treatment. Indeed, the progressive aneurysm occlusion depends on the FDS efficacy, which in turn depends on the hemodynamic changes caused by FDS placement within the aneurysm [5].

It is widely recognized that the evolution of intracranial aneurysm is linked to the hemodynamic environment characterizing the cerebral circulation. Hemodynamic stress can lead to endothelial dysfunction and activate inflammatory cytokines cascades, amplifying inflammatory processes [7]. In general, the shear stress on the artery wall can induce vascular remodeling and aneurysm growth and rupture [8]. Several studies suggest that wall shear stress (WSS) is one of the initiating factors for IA formation and responsible for its growth and rupture [9,10]. Thus, blood flow structures and wall shear stress distribution play a crucial role in the evolution of intracranial aneurysms. Interestingly, both high and low WSS can drive IA growth and rupture through different biological mechanisms, as temporal and spatial variations in WSS influence the rates at which endothelial cells are remodeled. Indeed, Meng and co-authors [10] proposed two different hemodynamic biologic pathways for the IA growth, which can be induced by low WSS and high oscillatory shear index (OSI), or by high WSS and positive WSS gradients, respectively. In particular, it should be emphasized that low WSS can lead to degenerative changes in the aneurysm wall and trigger the rupture in intracranial aneurysms through an inflammatory reaction [9,11,12,13].

Other hemodynamic indices that are associated with WSS and that are averaged over the cardiac cycle are significant for evaluating the evolution of IA, the risk of rupture, and the efficacy of endovascular treatment. Among them, time-averaged wall shear stress (TAWSS) has been found to be a fundamental parameter for understanding and predicting the growth and rupture of cerebral aneurysm [2,10,14]. In addition, the oscillatory shear index (OSI), the endothelial cell activation potential (ECAP), and the relative residence time (RRT) allow us to quantify the evolution of the aneurysm and the effect of flow diverter stent placement on intra-aneurysmal hemodynamics [5].

Numerical investigations allow us to determine the blood flow structures and to quantify the shear stresses that develop along the artery wall. Although these investigations have proven useful to predict the progression of aneurysmal disease, some issues regarding their use in the study of IA hemodynamics are not yet well clarified. In particular, most CFD simulation of the hemodynamics of intracranial aneurysms, even including FDS treatment, model blood as a Newtonian fluid despite the fact that blood typically exhibits a non-Newtonian behavior with shear-thinning characteristics [15,16,17,18,19]. Thus, the results of numerical simulations of blood flow in intracranial aneurysm could be highly dependent on the mathematical description of blood rheology adopted in solving the motion equations.

In general, the characteristics of blood flow depend on the blood viscosity, which in turn is influenced by the aggregation and deformability of red blood cells and additional parameters such as hematocrit and temperature [20]. Regarding cerebral arteries, there is no universal agreement on the best rheological model to represent the viscous properties of blood, and significant differences in intracranial hemodynamics were found due to the rheological model assumed in numerical investigation [21,22,23]. Furthermore, CFD simulations of intracranial aneurysm with presence of surface blebs showed high differences in TAWSS values between Newtonian and non-Newtonian modelling [24]. Obviously, particular attention is needed in regions of low wall shear stress that are associated with aneurysm rupture as the assumption of the rheological model can impact on hemodynamic forces within cerebral aneurysms and influence the numerical prediction of aneurysm rupture.

Regarding the choice of the rheological model, the assumption of the Newtonian model for blood can be accepted for blood flow in large arteries, which are characterized by high shear rates. However, in capillaries or small/medium arteries, the validity of the Newtonian hypothesis falls, and a non-Newtonian model should be adopted for blood viscosity. In particular, in intracranial arteries, evidence for non-Newtonian blood behavior was recently shown from Doppler ultrasonography measurements [25].

Taking into account the previous considerations, it is of interest to analyze to what extent the Newtonian assumption for blood affects the results of CFD simulations in cerebral aneurysm, i.e., if it substantially alters the values of the hemodynamic parameters that are essential to predict the risk of aneurysm rupture or the efficacy of FDS treatments. For this purpose, an accurate numerical investigation was performed to simulate the pulsatile blood flow inside a patient-specific intracranial aneurysm using constant Newtonian viscosity and non-Newtonian blood viscosity models (the Newtonian model, the Carreau model, and the Carreau–Yasuda model). In all these cases, the influence of the rheological blood model on the aneurysmal hemodynamics was analyzed both in the presence and the absence of FDS treatment of the aneurysm. For this aim, a flow diverter stent was appropriately reconstructed and virtually inserted in the patient-specific aneurysm, simulating real endovascular treatment.

To evaluate the influence of the blood rheological model on the hemodynamics of the intracranial aneurysm and the quantification of the efficacy of endovascular treatment, blood velocity distribution inside the aneurysm and the most important hemodynamic parameters were calculated before and after virtual endovascular treatment for each of the considered blood rheological models. The large difference found between Newtonian and non-Newtonian results suggests that the Newtonian assumption of constant blood viscosity should not be used to analyze the hemodynamics of intracranial aneurysm, especially in the presence of endovascular treatment.

## 2. Materials and Methods

### 2.1. Patient-Specific Model

**Figure 1:**
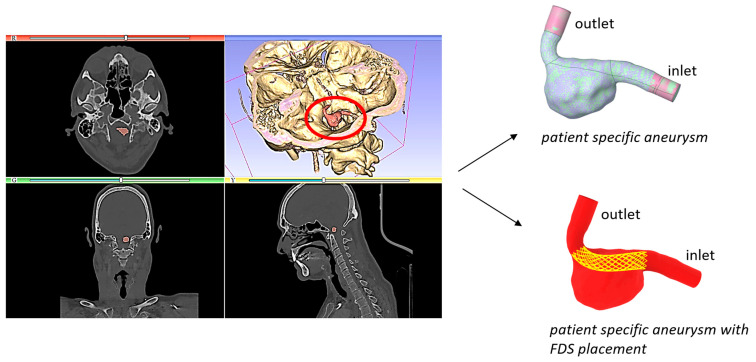
CTA image, aneurysm reconstructed model, and the model with virtual FDS placement. The red circle in the CTA image highlights the patient-specific aneurysm.

In this study, the hemodynamics in a patient-specific model of a saccular intracranial aneurysm was analyzed. The patient-specific geometry was previously reconstructed from Computed Tomography Angiography (CTA) data obtained from an Italian hospital [5]. The aneurysm was located in the vertebral artery. It was characterized by a maximum diameter of about 14 mm, and a wide neck of approximately 10 mm. Endovascular repair with deployment of a flow diverter stent was indicated for its treatment. The segmentation process of the medical images provided the geometric model that was further refined by eliminating imperfections and ramifications of the vessels not of interest. Extensions were inserted at the inlet and outlet of the model to allow the flow to fully develop before affecting the aneurysm. The segmentation and geometry refinement furnished the realistic and accurate fluid domain for numerical simulations (Figure 1).

To simulate clinical endovascular treatment, a flow diverter stent was reconstructed and virtually inserted into the patient-specific model. The geometric model of the stent that in the actual endovascular treatment would be implanted in the intracranial aneurysm was created using the commercial computer aided design (CAD) software Rhinoceros v.8. Since the flow diverter stents are braided tubular devices, the FDS was realized by superimposing circular wires onto the hypothetical healthy artery, i.e., the artery in the absence of aneurysm.

The main steps of the FDS reconstruction are the following. The patient-specific aneurysm model was imported into the Rhinoceros software and an initial FDS model was realized in the location where the clinician would deploy the stent. The initial and final sections of the stent were made proximal with the corresponding ones of the healthy artery. The diameter of the FDS wires was set equal to D = 100 µm. Then, the initial stent model, consisting of several circular wires wrapped onto a straight tube, was curved following the artery centerline. It should be underlined that following this methodology, the stent holes adapt to the curvature of the artery, unlike most stents made with a porous surface.

The porosity of the reconstructed FDS was calculated by the ratio of the void surface area to the total surface area of the device. Its value was approximately equal to 71%, in agreement with the optimal porosity value suggested in the literature [3,4].

Finally, a Boolean subtraction operation between the patient-specific aneurysm and the reconstructed stent was carried out to obtain the computational fluid domain for blood flow corresponding to the intracranial aneurysm with FDS implantation (Figure 1). This operation was performed using the Design Modeler software (Ansys Fluent 2022 R1).

### 2.2. Governing Equations and Numerical Setup

The pulsatile nature of blood flow was modelled using the mass and momentum conservation equations, which for an incompressible fluid and negligible gravity force are [26]:

(1) $$\nabla \cdot \vec{u}=0$$

(2) $$\rho \left(\frac{\partial \vec{u}}{\partial t}+\vec{u}\cdot \nabla \vec{u}\right)=-\nabla p+\nabla \cdot \tau$$

where *ρ* is the density of the fluid, $\vec{u}$ is the velocity vector, *p* the pressure, and τ is the deviatoric stress tensor. This tensor is related to the strain rate tensor D**,** D = (∇$\vec{u}\;$+ ∇$\vec{u\;}$^T^)/2, according to the relation:

$$\tau =2\;\mu (\dot{\gamma })\;D,$$

where μ is the dynamic viscosity of the fluid and $\dot{\gamma }$ is the shear rate.

The system of Equations (1) and (2), with the associated boundary and initial conditions, allowed us to determine the blood flow within the aneurysm.

In this study, a computational fluid dynamic (CFD) investigation was performed for modelling the blood flow in the patient-specific aneurysm under pulsatile flow conditions. Several experimental studies highlighted that blood exhibits non-Newtonian shear-thinning characteristics, i.e., its viscosity decreases with increasing shear rates, reaching a nearly constant value only for high shear rate values. To analyze the influence of the non-Newtonian rheological properties of blood on aneurysmal hemodynamics, a comparison was made between numerical results obtained using the Newtonian model with a constant blood viscosity *μ* = 0.0035 kg/(m·s) independent of the shear rate $\dot{\gamma }$, and those obtained with non-Newtonian models. In particular, the shear-thinning behavior of blood was modelled by using two of the most widely adopted non-Newtonian viscosity models, the Carreau model and the Carreau–Yasuda model.

In the Carreau model, the relationships for the dynamic viscosity *µ* is given by:

(3) $$\mu \left(\dot{\gamma }\right)=\mu_{\infty }+\left(\mu_{0}-\mu_{\infty }\right){\left[1+{\left(\lambda \dot{\gamma }\right)}^{2}\right]}^{\frac{n-1}{2}}$$

where *μ*_0_ = 0.056 kg/(m·s) is the viscosity at zero shear rate $\dot{\gamma }$, *μ*_∞_ = 0.0035 kg/(m·s) is the viscosity for an infinite shear rate, *λ* = 3.313 s is the relaxation time, and *n* = 0.3568 the power-law index [27].

In the Carreau–Yasuda model, viscosity *µ* tends to Newtonian viscosity at lower shear rates than in the Carreau case, and it is given by:

(4) $$\mu \left(\dot{\gamma }\right)=\mu_{\infty }+\left(\mu_{0}-\mu_{\infty }\right){\left[1+{\left(\lambda \dot{\gamma }\right)}^{a}\right]}^{\frac{n-1}{a}}$$

where *a*, *n*, and *λ* are constant parameters empirically determined. The quantities *a* and *n* are dimensionless, and *λ* has units of s. In particular, *μ*_0_ = 0.16 kg/(m·s), *μ*_∞_ = 0.0035 kg/(m·s), *λ =* 8.2 s, *a* = 0.64, and *n* = 0.2128 [28].

**Figure 2:**
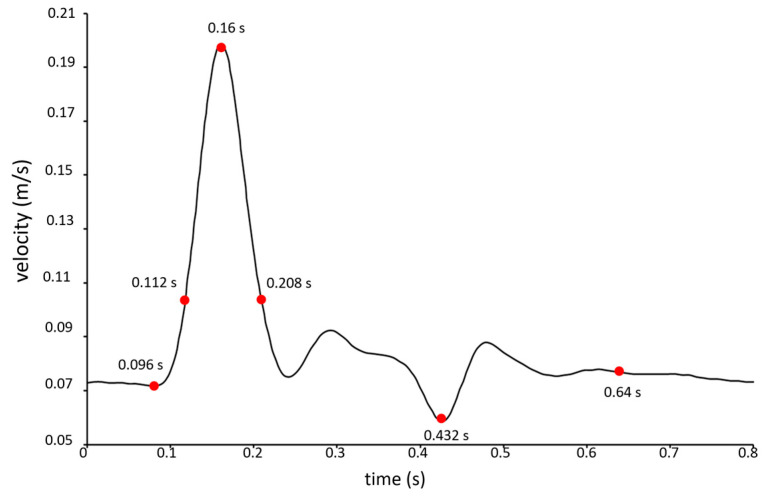
Pulsatile velocity waveform assigned at the inlet of the intracranial aneurysm model. The red points in the Figure indicate the instants of the cardiac cycle that were selected for the hemodynamic investigation.

The density of blood was assumed to be equal to 1060 kg/m^3^. A physiological velocity boundary condition was imposed at the inlet of the model (Figure 2) [29]. The period of the velocity waveform was equal to 0.8 s, the maximum velocity occurred at the systolic peak instant *t* = 0.16 s, and the minimum velocity was observed at the diastolic instant *t* = 0.432 s. Further significant instants were considered for describing hemodynamics inside the aneurysm, as illustrated in Figure 2. The instants *t* = 0.096 s, *t* = 0.112 s, and *t* = 0.208 s referred to systolic phase, and the instant *t* = 0.64 s referred to diastolic phase.

The Reynolds number Re = *ρ*UD/*μ* was defined with the blood density *ρ*, the blood dynamic viscosity *μ* = *μ*_∞_, the diameter D of the healthy artery at model inlet (D = 0.00427 m), and the velocity U assigned at the inlet. It had an averaged value of about Re_ave_ ≈ 134 (corresponding to the time-averaged velocity) and a maximum value Re_max_ ≈ 257, corresponding to the systolic peak velocity. Based on these values, a laminar blood flow was assumed.

On the wall of the patient-specific model, the no-slip boundary condition $\vec{u}=0$ was applied and the rigid wall assumption was made. Regarding this assumption, comments are furnished in Section 4. Furthermore, a pressure value *p* = 100 mmHg was assumed at the model outlet, as suggested in the literature [30].

The numerical investigation was performed using ANSYS Fluent.v.2022 R1 [31]. The semi-implicit method for pressure-linked equations (SIMPLE) was used, and an upwind scheme of the second order for the spatial discretization of momentum was adopted [32]. To reduce the effect of the initial numerical transient, CFD simulations were conducted over three cardiac cycles, and the third cycle only was considered for the analysis of intracranial hemodynamics.

The pre-operative IA model with no stent placement and the model with virtual endovascular treatment (i.e., IA model with FDS placement) were discretized into a large number of tetrahedral computational cells. The size of the mesh elements in the lumen was the same in both cases, but in the presence of the flow diverter stent inside the aneurysm, a refinement in the proximity of the devices was made.

Since the size of the mesh elements can greatly affect the accuracy of the numerical solutions, an investigation on the sensitivity of the numerical solution to the chosen mesh was performed to ensure that the results were not sensitive to the mesh size. For the mesh independence analysis, time averaged wall shear stress (TAWSS) values were monitored. The TAWSS parameter provides the average over the cardiac cycle of the magnitude of WSS vector and it is defined as:

$$TAWSS=\frac{1}{T}\int_{0}^{T}\left|\vec{WSS}\right|\;dt$$

where *T* is the period of the cardiac cycle and $\vec{WSS}$ the instantaneous WSS vector.

### 2.3. Mesh Sensitivity Analysis

Mesh independence analysis was performed both in the case of the patient-specific aneurysm without stent placement and in the case of the stent insertion inside the intracranial aneurysm. To ensure that the numerical results were not mesh-sensitive, TAWSS values on a curve located at the center of the aneurysm dilatation (middle curve) were compared for different mesh sizes. This parameter was selected for the mesh sensitivity analysis, as its values are very sensitive to the mesh size.

**Figure 3:**
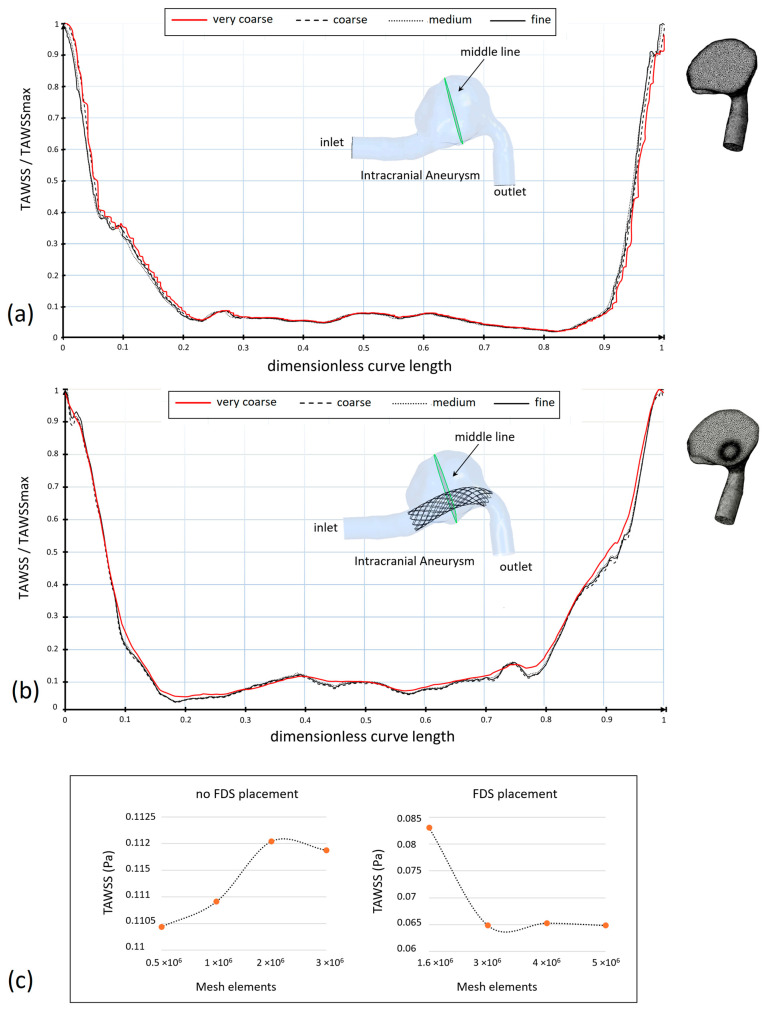
Dimensionless TAWSS along the selected middle curve in the patient-specific aneurysm for four different mesh sizes (very coarse, coarse, medium, fine). (**a**) Refers to the aneurysm with no FDS placement and with number of mesh elements approximately equal to 0.5 × 10^6^, 1 × 10^6^, 2 × 10^6^, and 3 × 10^6^. (**b**) Refers to the aneurysm with FDS placement and number of mesh elements approximately equal to 1.6 × 10^6^, 3 × 10^6^, 4 × 10^6^, and 5 × 10^6^. (**c**) Shows the plots of the mesh convergence. All values refer to the Carreau rheological model.

Figure 3 shows the TAWSS values along the selected curve for four mesh sizes (very coarse, coarse, medium, fine), and the plots of mesh convergence (Figure 3a–c, respectively). Both the case of no stent placement and the case of stent insertion are shown. Although there is a general agreement among TAWSS profiles, it can be observed that the difference in the TAWSS profiles reduces as the number of mesh elements increases.

To show this result more clearly, the values of the TAWSS averaged on the middle curve of the aneurysm are shown in Table 1 for the different meshes. Only a small percentage variation of less than 1% between the TAWSS obtained with the highest meshes was found, both in the presence and the absence of FDS placement. This result suggested the choice of the optimal meshes which approximately have 3 × 10^6^ elements in the absence of flow diverter stent and 5 × 10^6^ elements in the presence of the device. These values were obtained using a tetrahedral element size equal to 0.12 mm in the lumen in both cases, and 0.07 mm in the proximity of the flow diverter stent if inserted.

Another factor that may affect the computational results is the size of the time step used in numerical simulations. The smaller the value of the time step used, the greater the precision of the numerical solution. However, as the value of the time step increases, the calculation time increases significantly. Therefore, a preliminary study was performed to analyze the influence of the time step size on the results of hemodynamic investigations.

Three time step values (0.016 s, 0.008 s, and 0.004 s) were tested for simulating the blood flow in the IA patient-specific model without stent placement, with a mesh of approximately 3 × 10^6^ elements. For the three considered time steps, the WSS at systolic peak and TAWSS, both of them averaged on the aneurysmal wall, were compared to determine the optimal time step. The difference between results obtained with the two smallest time steps was less than 0.7% for WSS and less than 1.5% for TAWSS. Thus, the time step size 0.008 s was selected for the numerical simulations, and 200 iterations were performed for each time step. Furthermore, the convergence criteria for the residuals of velocity components and continuity were set to 10^−5^ at each time-step.

### 2.4. Hemodynamic Parameters

Time-averaged hemodynamic parameters related to WSS are useful to understand the complex hemodynamic fields that characterize intracranial hemodynamics. Furthermore, they were revealed to be essential for describing the effect of the blood flow on the aneurysm evolution after FDS implantation. These include the TAWSS previously defined, and the following fundamental parameters: OSI, ECAP, and RRT. All these hemodynamic indicators were determined and analyzed in this work.

The oscillatory shear index is furnished by the following relationship:

$$OSI=\frac{1}{2}\left(1-\frac{\left|\int_{0}^{T}\vec{WSS}\;dt\right|}{\int_{0}^{T}\left|\vec{WSS}\right|\;dt}\right)$$

OSI is a non-dimensional parameter whose value ranges from 0 to 0.5 and accounts for directional changes of the WSS vector during the cardiac cycle with respect to the dominant direction of the flow [33]. Zero OSI value corresponds to unidirectional shear stress, and it is related to a healthy condition. On the contrary, a high OSI value is believed to induce an inflammatory response on the artery wall [34,35].

The endothelial cell activation potential ECAP correlates TAWSS and OSI values:

ECAP = OSI/TAWSS

This parameter is frequently employed to characterize the ‘thrombogenic susceptibility’ of the artery wall. A high OSI value and low TAWSS value determine high ECAP values that are associated with conditions of endothelial susceptibility [36,37].

Finally, the RRT parameter allows for the evaluation of the residence time of particles near the vessel wall [38]:

$$RRT=\frac{1}{\left(1-2\cdot OSI\right)\cdot TAWSS}=\frac{1}{1/T\left|\int_{0}^{T}\vec{WSS}\;dt\right|}$$

This parameter is fundamental in the study of the aneurysmal hemodynamics, as the evaluation of the relative residence time of particles within the aneurysm allows us to identify the possibility of thrombus formation and consequent occlusion of the aneurysm [39].

## 3. Results

Numerical simulations of the pulsatile blood flow within the intracranial aneurysm were performed with and without placement of the flow diverter stent inside the patient-specific model. In particular, different models for blood viscosity were used to evaluate the influence of rheological model on the intracranial hemodynamics. Numerical results included 2D and 3D streamlines, velocity contours and the hemodynamic parameters TAWSS, OSI, ECAP, and RRT that are significant for the aneurysm progression and the FDS effectiveness. Furthermore, due to the crucial role of WSS on the growth and possible rupture of intracranial aneurysm, WSS at selected instants of the cardiac cycle were calculated.

### 3.1. The Effect of the Rheological Model on WSS

**Figure 4:**
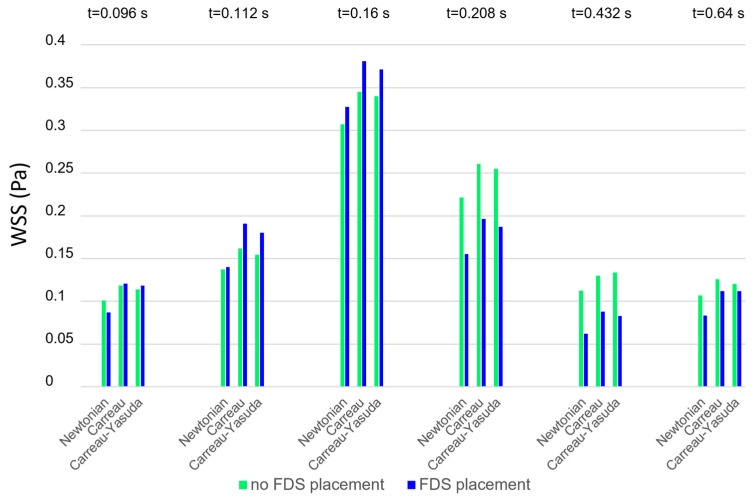
WSS values averaged on the wall of the aneurysmal dilatation at selected instants of the cardiac cycle obtained using different rheological models.

Figure 4 shows the instantaneous WSS values averaged on the aneurysm wall obtained using different blood rheological models in the numerical simulations (Newtonian model, Carreau model, and Carreau–Yasuda model). The values refer to the instants of the cardiac cycle shown in Figure 2, in the absence and presence of FDS treatment (green and blue bars, respectively). For any rheological model, the presence of stents (blue bars) increases the WSS values in the acceleration phase of the cardiac cycle and lowers them in the deceleration phase, which is associated with slow recirculating blood motion. It is interesting to note that at any instant of the cardiac cycle the Newtonian assumption for blood viscosity determines WSS values lower than the non-Newtonian ones. This result is confirmed in both the presence and absence of stent placement inside the aneurysm. As will be seen later, a similar result also applies to the TAWSS values.

### 3.2. The Effect of the Rheological Model on Intra-Aneurysm Blood Flow

**Figure 5:**
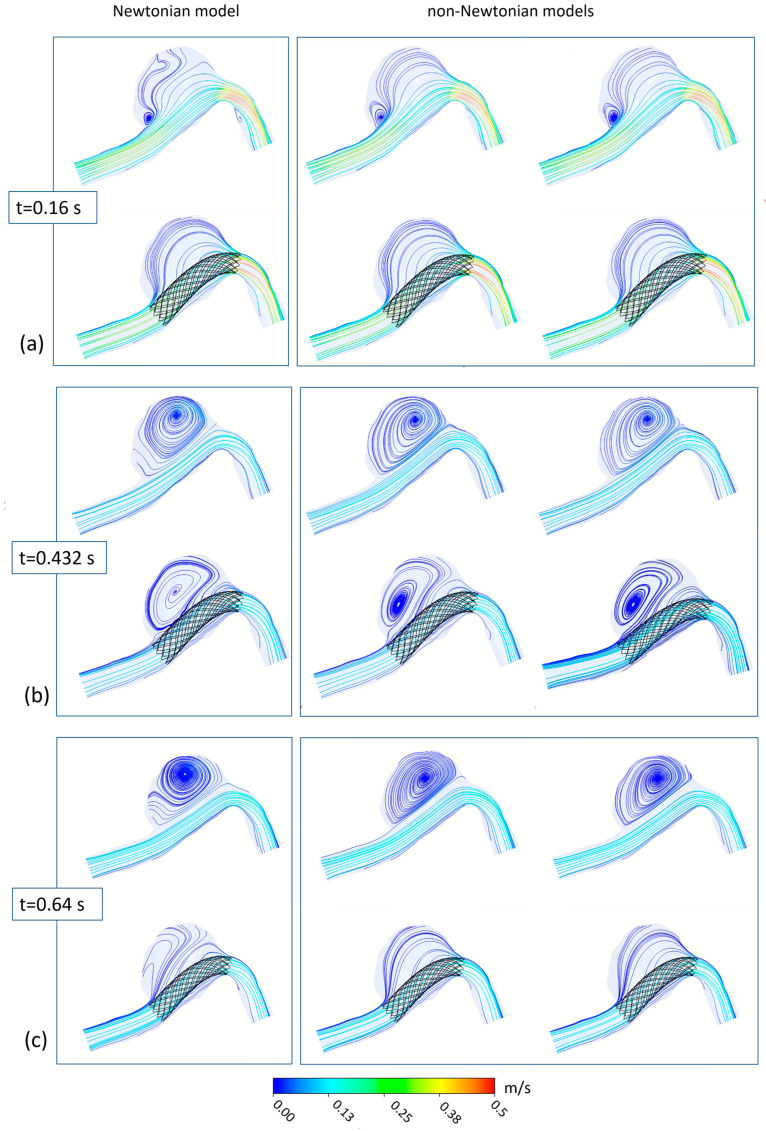
2D streamlines in the patient-specific aneurysm with and without FDS insertion obtained with different rheological models for blood: (**a**) refers to the systolic velocity peak (*t* = 0.16 s), (**b**) refers to the diastolic velocity minimum (*t* = 0.432 s), (**c**) refers to the late diastole (*t* = 0.64 s). The first column shows the results obtained when adopting the Newtonian model in the numerical simulation; the second and third columns refer to the Carreau and Carreau–Yasuda models, respectively.

Figure 5 shows the 2D velocity streamlines on a longitudinal section of the patient-specific aneurysm, with and without FDS insertion. The streamlines were obtained with different viscosity models and are shown in correspondence to the main instants of the cardiac cycle: the systolic peak instant, corresponding to the maximum value of inlet velocity (*t* = 0.16 s), the diastolic instant of minimum inlet velocity (*t* = 0.432 s), and a typical instant of the late-diastole phase (*t* = 0.64 s). In all cases, the same velocity range and the same number of streamlines colored with the velocity magnitude are used to visualize the instantaneous flow structures. The results shown in Figure 5 highlight the influence of the rheological model of blood on the intracranial hemodynamics and the effect of FDS treatment on the intra-aneurysmal blood flow. The first column shows the results obtained adopting the Newtonian rheological model in the numerical simulations, and the second and third columns refer to the assumption of the Carreau and the Carreau–Yasuda model, respectively. For each of the considered instants of the cardiac cycle, the first row in the figure refers to the case of absence of endovascular treatment; the second row shows the streamlines distribution in the presence of FDS placement inside the aneurysm.

The main observation about these results is that the velocity streamlines obtained using the Newtonian model differ substantially from those obtained with the non-Newtonian models, which, on the contrary, are quite similar to each other.

Furthermore, the effect of blood flow redirection into the parent artery due to the insertion of flow diverter stent in the aneurysm model is clearly shown. In particular, the presence of the stent determines a substantial reduction in blood recirculation and velocity magnitude inside the aneurysm, significantly modifying intracranial hemodynamics.

Referring to the case of absence of endovascular treatment, the following can easily be observed. At the systolic peak instant *t* = 0.16 s, a small recirculation area in the proximal side of the aneurysmal dilatation is detected. However, also a secondary vortex appears if the Newtonian model for the blood rheology is used. At the diastolic minimum instant *t* = 0.432 s, the velocity of the blood stream in the parent artery decreases considerably, reaching a minimum value. The small vortex observed inside the aneurysm at the systolic peak instant widens. A large counter-clockwise recirculating region appears inside the aneurysm characterized by slow flow entering the dilatation distally and flowing back along the aneurysm wall. Even at this instant, the Newtonian results differ substantially from the non-Newtonian ones. Finally, at the instant *t* = 0.64 s that characterizes the last phase of the cardiac cycle, the low velocity of the blood stream allows for the maintenance of the large recirculating region inside the aneurysm. Even at this instant, the Newtonian assumption for the rheological model of blood determines a significant alteration in the blood flow pattern with respect to that obtained with non-Newtonian models, which, on the contrary, furnish results that are quite similar to each other.

The difference between Newtonian and non-Newtonian results is confirmed in the presence of the FDS treatment, where the effect of the Newtonian assumption on the blood flow is even more evident, and more significant changes in the velocity distributions alter the blood flow especially in the diastolic phase of the cardiac cycle.

Obviously, the correct knowledge of the blood flow pattern within the aneurysm is fundamental because recirculation regions give rise to non-physiological shear stress on the luminal surface of the aneurysm, which in turn is correlated with the vascular wall remodeling and aneurysm development or occlusion [40].

### 3.3. The Effect of the Rheological Model on Hemodynamic Parameters

Although 2D and 3D streamlines and velocity contours allow for the investigation of the complex velocity field that characterizes the intracranial aneurysm, further hemodynamic parameters are generally used for a better understanding of the aneurysm evolution. Recently, CFD simulations of blood flow in intracranial aneurysm have significantly improved the understanding of intracranial hemodynamics. However, since there is no general agreement on the role of high or low WSS on the growth and rupture of intracranial aneurysm [10], the evaluation of additional parameters, such as TAWSS, OSI, ECAP, and RRT, was found to be fundamental to a more thorough analysis of the aneurysm evolution and quantification of the effectiveness of FDS. Nevertheless, the values of these hemodynamic parameters could be dependent on the viscosity model adopted to simulate the blood flow in the numerical investigation. Taking into account the previous considerations, the effect of the rheological model adopted for blood on the mentioned time-averaged parameters was analyzed.

**Figure 6:**
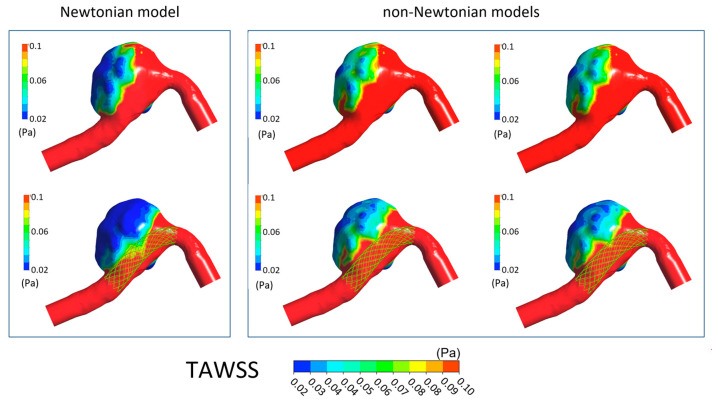
TAWSS contours in the absence of FDS placement (**first row**) and in the presence of virtual FDS placement (**second row**). First box shows results obtained adopting the Newtonian model; second box shows those obtained with the Carreau and Carreau–Yasuda non-Newtonian models (I and II column, respectively).

**Figure 7:**
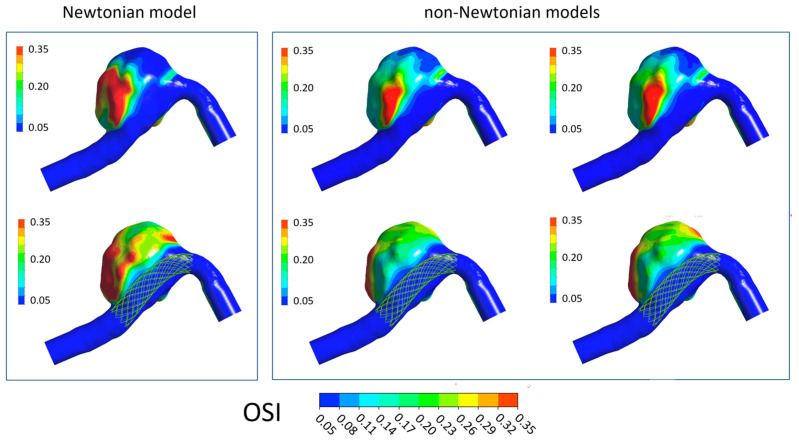
OSI contours in the absence of FDS placement (**first row**) and in the presence of virtual FDS placement (**second row**). First box shows results obtained adopting the Newtonian model; second box shows those obtained with the Carreau and Carreau–Yasuda non-Newtonian models (I and II column, respectively).

**Figure 8:**
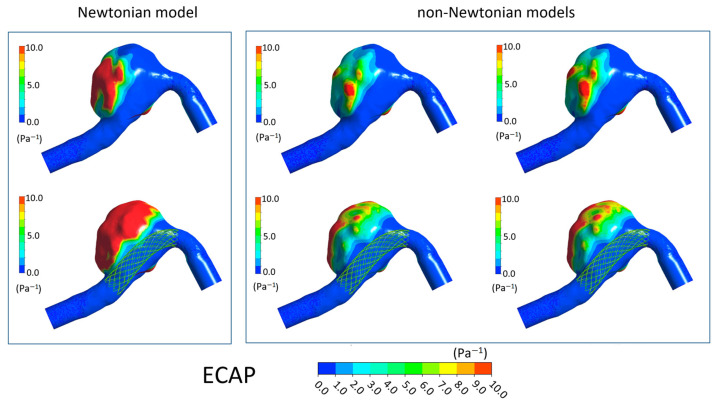
ECAP contours in the absence of FDS placement (**first row**) and in the presence of virtual FDS placement (**second row**). First box shows results obtained adopting the Newtonian model; second box shows those obtained with the Carreau and Carreau–Yasuda non-Newtonian models (I and II column, respectively).

**Figure 9:**
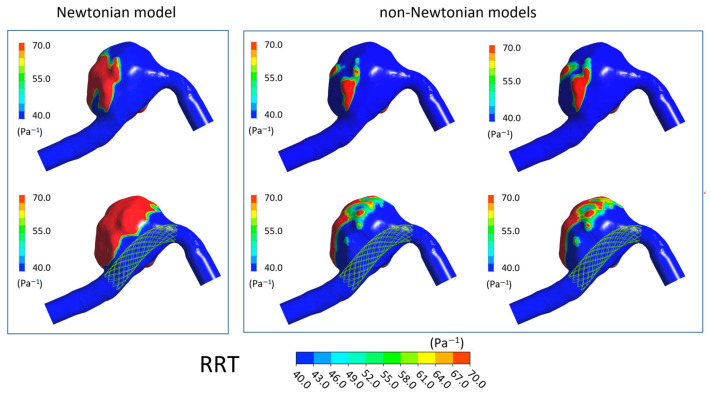
RRT contours in the absence of FDS placement (**first row**) and in the presence of virtual FDS placement (**second row**). First box shows results obtained adopting the Newtonian model; second box shows those obtained with the Carreau and Carreau–Yasuda non-Newtonian models (I and II column, respectively).

In Figure 6, Figure 7, Figure 8 and Figure 9 the TAWSS, OSI, ECAP, and RRT contours on the luminal surfaces of the patient-specific aneurysm in the absence and presence of virtual FDS placement are shown. The spatial distribution of the hemodynamic parameters on the aneurysm wall was calculated both adopting the Newtonian model for blood and the non-Newtonian Carreau and Carreau–Yasuda models.

It can be seen that the presence of FDS determines a global decrease in TAWSS values within the aneurysms. However, results obtained when adopting the Newtonian hypothesis for blood viscosity differ significantly from that calculated with non-Newtonian models, which instead provide reasonably similar distributions for all hemodynamic parameters. In particular, the blood Newtonian assumption provides significantly lower TAWSS values both in the absence and presence of FDS placement compared to that obtained with non-Newtonian models. Furthermore, more extensive regions of low TAWSS are observed (Figure 6).

On the contrary, the OSI, ECAP, and RRT values increase in the presence of FDS. However, values obtained with the Newtonian model are higher than those calculated with the non-Newtonian models, and larger regions with high values of these parameters are found on the aneurysmal wall (Figure 7, Figure 8 and Figure 9). Once again, in the cases of non-Newtonian models, the contour maps of the parameters are reasonably similar to each other. These observations are valid both in the absence and presence of FDS placement.

**Figure 10:**
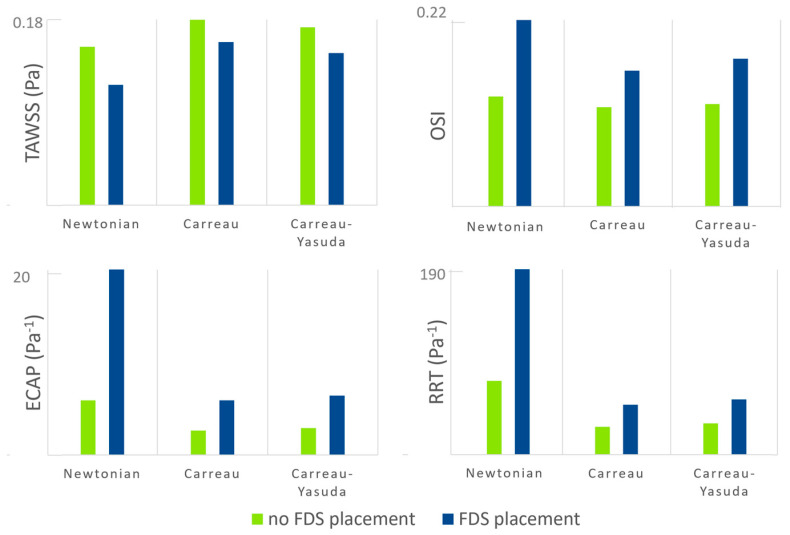
TAWSS OSI, ECAP, and RRT values averaged on the aneurysmal surface in both the presence and absence of endovascular treatment (green bars and blue bars, respectively).

In addition, to analyze the spatial distribution of the hemodynamic parameters, some values derived from these indices can help quantify the effect of the blood rheological model on intra-aneurysm hemodynamics. For this purpose, the spatial average on the aneurysmal surface of the time-averaged quantities TAWSS, OSI, ECAP, and RRT was calculated both in the presence and absence of endovascular treatment. The results are shown in Figure 10. The discrepancy between the values of the hemodynamic parameters obtained with the Newtonian hypothesis and those resulting from the non-Newtonian assumptions is clearly highlighted in the Figure. In particular, the Newtonian model provides significantly lower TAWSS and higher OSI, ECAP, and RRT on the aneurysm wall than the non-Newtonian ones both in the presence and absence of endovascular treatment.

It is possible to note that the deviation between Newtonian and non-Newtonian results is much larger in the ECAP and RRT values than in the TAWSS and OSI values. Interestingly, the difference between Newtonian and non-Newtonian ECAP and RRT increases significantly in the presence of stent placement within the intracranial aneurysm. Since the ECAP values characterize the ‘thrombogenic susceptibility’ of the artery wall [36] and the RRT values quantify the propensity to produce blood thrombosis into the aneurysm [38], these results mean that numerical simulations performed using the Newtonian hypothesis provide a much more optimistic prediction of the effectiveness of FDS and consequent healing of the aneurysmal disease.

The extremely large difference between Newtonian and non-Newtonian RRT values and the well-kwon non-Newtonian blood properties in small arteries, especially in cerebral ones, indicate the need of a non-Newtonian rheological model to adequately simulate blood flow in intracranial aneurysm, especially in the presence of endovascular treatment.

### 3.4. The Effect of FDS Placement on Intra-Aneurysm Blood Flow

Lastly, to better highlight the effect of flow diverter stent placement within the aneurysm, the blood flow in the intracranial aneurysm was numerically simulated using the non-Newtonian Carreau model.

**Figure 11:**
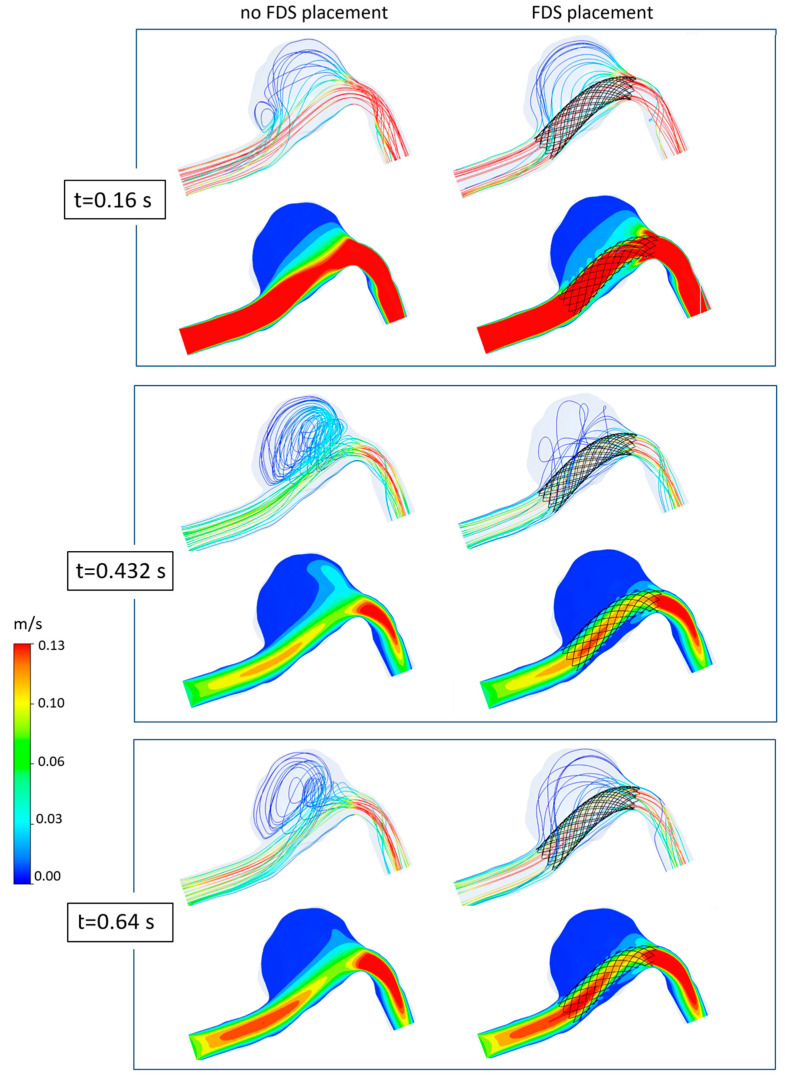
3D streamlines and velocity magnitude contours on a longitudinal section of the aneurysm at the systolic peak instant *t* = 0.16 s, the diastolic minimum instant *t* = 0.432 s and the late diastole instant *t* = 0.64 s obtained with the Carreau rheological models. First column refers to the case of no FDS placement; second column shows results in the presence of FDS within the aneurysm. The streamlines are colored by the local velocity magnitude.

In Figure 11, the 3D streamline distributions and velocity contours within the aneurysmal dilatation at different instants are shown both in the case of no stent placement and in the case of stent insertion. For simplicity, only the three main instants of the cardiac cycle are shown, and a velocity range smaller than that used in Figure 5 is adopted to also highlight the streamlines with very low velocity. Obviously, the same number of streamlines is displayed both in the presence and absence of FDS within the aneurysm.

It can be seen that the presence of the flow diverter stent significantly modifies the intracranial hemodynamics, reducing inflow, velocity, and recirculation within the aneurysm. In particular, the effectiveness of the device in suppressing or weakening the recirculation regions is well-depicted by the 3D streamlines (first rows in Figure 11). Furthermore, the velocity contour maps clearly highlight the beneficial effect of the stent in redirecting blood flow into the parent artery and reducing the magnitude of velocity in the aneurysm (second rows in Figure 11). In particular, the placement of FDS determines a global decrease in the velocity values inside the dome at all instants of the cardiac cycle. At the instants *t* = 0.432 s and *t* = 0.64 s that characterize the diastolic phase, the reduction in intra-aneurysmal velocity of blood flow is particularly evident, and mainly affects the distal region of the aneurysmal dilatation. Conversely, at the systolic peak *t* = 0.16 s corresponding to the fastest incoming flow, the velocity reduction is more uniform within the dilatation, and affects both the distal and the proximal regions of the aneurysm.

At last, it was observed that the aneurysmal pressure values remained almost unchanged after the placement of FDS, according to numerical and in vivo results [18,41,42]. In particular, at the systolic peak instant, only a small increase of 0.18 mmHg was found in the presence of the stent placement.

**Figure 12:**
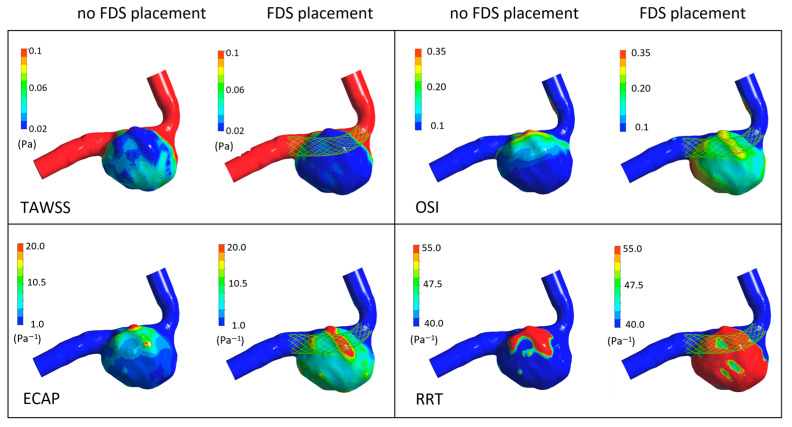
TAWSS, OSI, ECAP, and RRT contours in the absence of FDS placement and in the presence of virtual FDS placement obtained adopting the non-Newtonian Carreau model.

Furthermore, Figure 12 shows the time-averaged hemodynamic parameters, TAWSS, OSI, ECAP, and RRT, which clearly highlight the effect of FDS placement within the aneurysm. It can be observed that the stent placement determines a consistent decrease in the values of the time-averaged wall shear stress, TAWSS, and an increase in the values of the oscillatory shear index, OSI, and Endothelial Cell Activation Potential, ECAP, on the aneurysmal surface. In particular, the ECAP parameter was analyzed since it combines the effects of OSI and TAWSS, taking into account the level of the shear and its oscillatory character [37].

Even more important is the fact that the presence of stent causes a very high increase in the relative residence time, RRT, values, which suggest thrombotic occlusion of the intracranial aneurysm [39] and parent artery reconstruction, i.e., healing of the aneurysmal disease.

## 4. Study Limitations

Some assumptions were adopted in this study. Due to the lack of the patient-specific inlet velocity waveform, a typical pulse of the vertebral artery was employed. However, some of the literature findings indicate that inlet waveform has minimal effects on WSS and OSI distribution, although it influences the OSI magnitude [43]. In addition, a recent study on 156 IAs highlighted that patient-specific inflow waveform may not be necessary for predicting rupture risk since the same significant parameters associated with rupture were detected using patient-specific or generalized inflow conditions [44].

Furthermore, the rigid wall assumption may affect the results of the study, although it is commonly adopted in CFD investigations on the IA hemodynamics. In order to justify this hypothesis, it can be considered that the loss of elastic lamina and the substantial variability in collagen architecture detected in intracranial aneurysms determines a reduced wall compliance and can support this assumption [45,46]. In addition, the use of fluid–structure interaction models, which account for wall compliance, can in turn introduce numerical errors if patient-specific properties of the arterial wall are unknown. In particular, quantities such as aneurysmal wall stiffness, thrombus properties, and local thickness of the aneurysm wall are generally unknown, and the lack of the real values of these quantities can determine sensible changes in the hemodynamic parameters. For instance, the assumption of a uniform wall thickness in fluid–structure simulations can lead to strong differences in the wall stress distribution and the stress values at the rupture site of the intracranial aneurysm compared to those obtained when accounting for non-uniform wall thickness distribution [47].

Future studies will address these limitations. In particular, it would be interesting to analyze the effect of flexible arterial walls and the deformability of platelets and red blood cells.

## 5. Discussion and Conclusions

In recent years, endovascular intervention with flow diverter stent (FDS) deployment has been increasingly adopted for the treatment of cerebral aneurysm, being a minimally invasive technique with low complication rates and high efficacy. Having reliable information on the efficacy of the FDS treatment before the actual stent placement may be fundamental for the treatment planning or the need for surgery. In this context, computational fluid dynamics investigations on patient-specific aneurysms have proven to be a fundamental tool. However, it should be noted that most CFD investigations on the hemodynamics in cerebral aneurysms and FDS treatment adopt a Newtonian behavior for blood.

The aim of this study was to evaluate the influence of the blood rheological model on the intracranial aneurysm hemodynamics and the efficacy of endovascular treatment. Different rheological models for blood viscosity were used to numerically solve the equation of the pulsatile motion in a patient-specific intracranial aneurysm. In all cases, the influence of the rheological blood model on aneurysmal hemodynamics was analyzed both in the presence and absence of FDS treatment.

The results of the numerical investigation highlighted that the placement of FDS reduces blood recirculation and intra-aneurysmal velocity, redirecting the blood flow away from the aneurysm into the parent artery. Furthermore, a reduction in the time-averaged wall shear stress (TAWSS) values and an increase in the oscillatory shear index (OSI), endothelial cell activation potential (ECAP), and relative residence time (RRT) values were found. In particular, presence of FDS causes a high increase in RRT values, determining a favorable hemodynamic environment to promote thrombus formation and occlusion of the aneurysm.

However, numerical simulation of blood flow was found to be heavily dependent on the mathematical description of blood rheology. The velocity distributions obtained using the Newtonian model differ substantially from those obtained with the non-Newtonian models, which, on the contrary, provide results quite similar to each other. Furthermore, the Newtonian model provides significantly lower TAWSS on the aneurysmal wall, and higher OSI, ECAP, and RRT than the non-Newtonian ones, both in the presence and absence of endovascular treatment.

Interestingly, in the presence of stent placement, the Newtonian model largely overestimated the ECAP and RRT values with respect to the non-Newtonian models, i.e., the assumption of Newtonian blood viscosity provides an unrealistic overestimation of blood thrombosis within the aneurysm and effectiveness of FDS treatment. This overly optimistic prediction of healing suggested by the Newtonian model could be misleading for the choice of endovascular or surgical treatment to be adopted. Indeed, it is well known that the Newtonian model does not take into account the intrinsic rheological characteristics of blood, especially in the cerebral arteries. However, it is the large difference found between Newtonian and non-Newtonian results that indicates that the blood Newtonian assumption should not be used in the study of the hemodynamics of intracranial aneurysm, especially in the presence of endovascular treatments.

## Figures and Tables

**Table 1:** TAWSS averaged on the middle curve of the aneurysm, for different numbers of mesh elements (values refer to Carreau rheological model).

No FDS Placement			FDS Placement		
**Elements** (approximate number)	**TAWSS** (Pa)	**Variation** (%)	**Elements** (approximate number)	**TAWSS** (Pa)	**Variation** (%)
0.5 × 10^6^	0.110436		1.6 × 10^6^	0.0830499	
1 × 10^6^	0.110909	0.43	3 × 10^6^	0.064836	−21.93
2 × 10^6^	0.112039	1.09	4 × 10^6^	0.065274	0.68
3 × 10^6^	0.111874	−0.15	5 × 10^6^	0.064842	0.66

## Data Availability

The original contributions presented in the study are included in the article, further inquiries can be directed to the corresponding author.
